# Small incision basilic vein transposition technique: A good alternative to standard method

**DOI:** 10.4103/0970-1591.60466

**Published:** 2010

**Authors:** Muthu Veeramani, Jigish Vyas, Ravindra Sabnis, Mahesh Desai

**Affiliations:** Department of Urology, Muljibhai Patel Urological Hospital, Nadiad, Gugarat - 387 001, India

**Keywords:** Basilic vein, transposition, vascular access

## Abstract

End-stage renal disease is a significant health problem. The primary use of the autogenous arteriovenous access is recommended by NKF-DOQI (National Kidney Foundation-Dialysis Outcomes Quality Initiative) guidelines. Though basilic vein transposition is well established in multiple failed fistulae's and obese patients, it requires large incision and morbidities like edema and infection. To avoid such compilations we, at our institution, adopted a small incision technique using two small 3-4 cm incisions. This method is inspired by videoendoscopic minimally invasive method used to dissect the basilic vein, thus avoiding extensive dissection and related morbidities.

## INTRODUCTION

End-stage renal disease (ESRD) is a significant public health problem.[1] During the past few decades, there is increasing prevalence of patients requiring hemodialysis. The primary use of autogenous arteriovenous access for chronic hemodialysis is recommended by NKF-DOQI guidelines. The Brescia-Cimino wrist fistula remains the procedure of choice, followed by the brachiocephalic arteriovenous fistula (BCAVF) formed at the elbow.[2] Basilic vein transposition (BVT) was first described in 1976 and has been increasingly accepted as a viable option for secondary or tertiary vascular access.[3] Fistulas created with a transposed basilic vein sutured end to side to the brachial artery have been shown to be the most reliable and dependable secondary vascular access procedure reported for chronic hemodialysis. Primary patency rates for the first and second year range from 80 to 90% and 74 to 86%, respectively, with a long term patency of 70% at eight years reported in a large series.[4] Basilic vein is long, lies deep and generally free of puncture with a relatively large diameter and higher venous flow with high patency and maturation rates. It matures early and provides a longer conduit for dialysis. But BVT is time consuming and technically challenging procedure with significant perioperative morbidity due to long incisions and extensive surgical dissection.[5]

## CONVENTIONAL BASILIC VEIN TRANSPOSITION (BVT)

**Figure 1:**
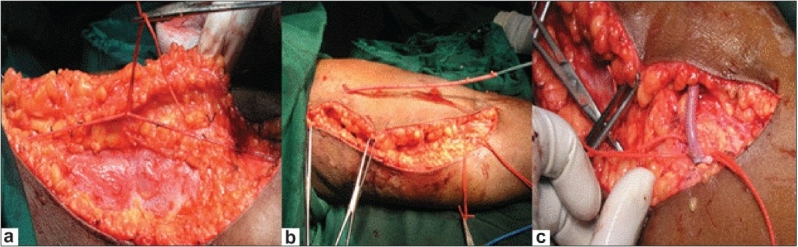
(a) Large incision for formation of traditional BVT; (b) Exteriorization of basilic vein; (c) End to side brachio basilic transposition

Conventional BVT [Figure 1] requires long incision over the medial aspect of the arm. After dissecting the basilic vein up to axillary vein, it is cut in the cubital fossa and transposed into the subcutaneous tissue by multiple small incisions. End to side basilic vein brachial artery anastmosis is done.

**Figure 2:**
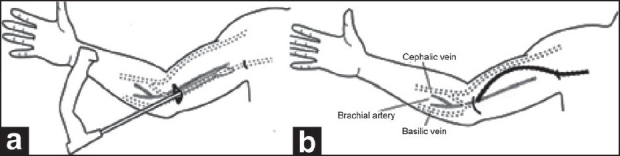
Video endoscopic technique (a) Creation of working space; (b) Basilic vein transposition

We performed the small incision technique at our institute. It requires two small 3-4 cm incisions to dissect the basilic vein. It is inspired by the minimally invasive technique of basilic vein dissection done by videoendoscope.[6] In this technique, after creating an operative working space, the basilic vein was liberated along its length by endovein harvesting dissector, by ligating and dividing the visualized venous tributaries [Figure 2]. Chemla *et al*. describe this technique and prospectively compare BVT with arterio venous graft; to the best of our knowledge the outcome of the technique is not described.[7]

## SMALL INCISION TECHNIQUE

Basilic vein is dissected after a 3 cm incision over the medial aspect of the cubital fossa [Figure 3] and dissecting towards the arm. After lifting the skin with the hook retractors the tributaries of the vein are cut between ligatures. Once it is felt that further dissection was not possible from this incision, another 3-4 cm longitudinal incision in the medial aspect of the upper arm and dissection of the basilic vein continued proximally. Once dissection is completed up to the axillary vein the bsasilic vein is divided at elbow and transposed in the anterior surface of the arm in the subcutaneous plane and brachio basilic side to end anastomosis is performed [Figure 3].

**Figure 3a:**
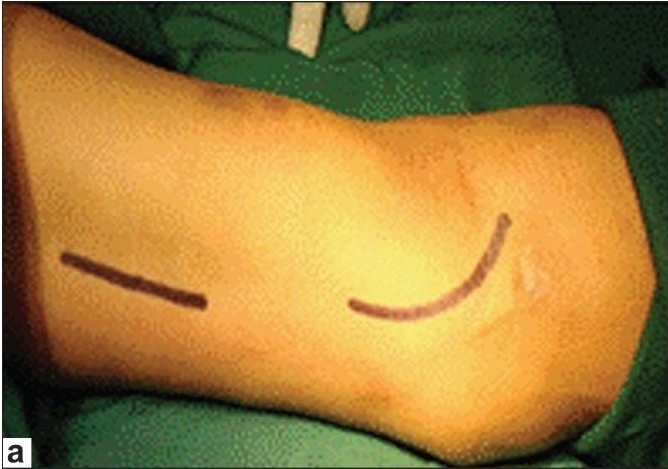
(a) Marking of incisions

**Figure 3b:**
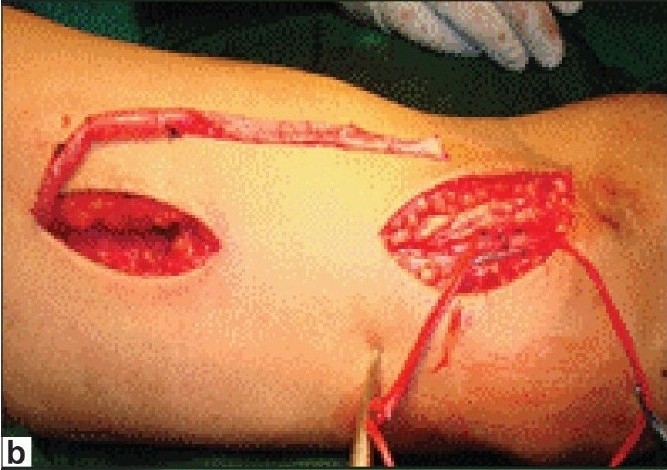
Exteriorization of basilic vein

**Figure 3c:**
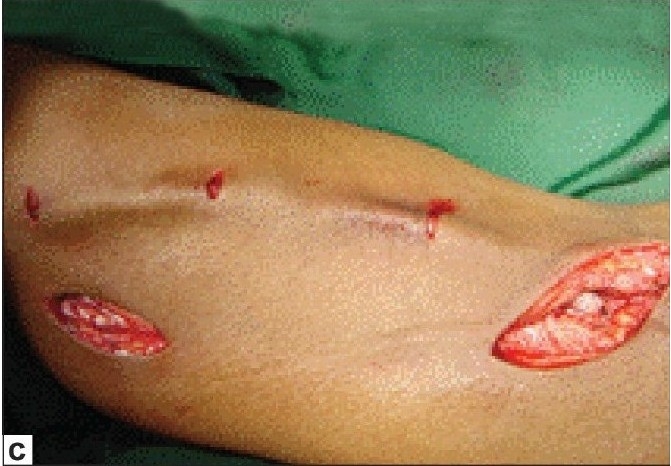
Brachio basilic transposition

## RESULTS

Fourteen cases were treated with this technique from March 2006 to June 2008. The mean age was 58.2 years, nine were males. At one year follow-up, mean primary patency rates was 78.57% ( = 11), secondary patency rate was 85.71% ( = 12). Maturation time at four weeks was 71.42% ( = 10). Primary failure was 21.3% ( = 3) secondary to poor maturation. One patient died due to cardiac arrest. Two (14.2%) patients with arm edema and two with infection were treated with arm elevation and antibiotics. Three patients required re exploration due to post procedure bleeding, hematoma, and thrombosis.

## DISCUSSION

Autologous arteriovenous hemodialysis access has been the “gold standard” for patients needing hemodialysis for 30 years.[8] The brachiobasilic arteriovenous fistula is increasingly the access procedure of choice when a superficial arm vein is unavailable. Reported long term cumulative patency rates are in the range of 54-90%, 38-82% and 43-57% at 1, 2 and 3 years respectively.[9] The reported complication rate for BVT remains high at 47-71%.[10] One year follow-up results and complications in our study matched these figures. Pre-operative assessment of basilic vein quality and caliber using duplex ultrasound has been increasingly advocated as a way of improving fistula outcome, particularly in terms of technical success rate.[11]

## CONCLUSION

The need for reliable, long-term hemodialysis vascular access remains critical. BVT is the most durable hemodialysis access procedure. The small incision technique for exteriorization of the basilic vein has an added advantage of small incision, less arm edema and comparable complication rate to that of standard technique. More prospective randomized studies are required to validate this small incision technique.
